# Psoas Major Swelling Grade Affects the Clinical Outcomes after OLIF: A Retrospective Study of 89 Patients

**DOI:** 10.1111/os.13774

**Published:** 2023-07-05

**Authors:** Zefeng Song, Wanyan Chen, Guangye Zhu, Xingda Chen, Zelin Zhou, Peng Zhang, Shaohao Lin, Xiaowen Wang, Xiang Yu, Hui Ren, De Liang, Jianchao Cui, Xiaobing Jiang, Jingjing Tang

**Affiliations:** ^1^ First Clinical Medical College Guangzhou University of Chinese Medicine Guangzhou China; ^2^ Department of Spinal Surgery The First Affiliated Hospital of Guangzhou University of Chinese Medicine Guangzhou China

**Keywords:** Minimally Invasive Fusion, Oblique Lumbar Interbody Fusion, Psoas Major, Psoas Major Swelling Grade

## Abstract

**Objects:**

Oblique lumbar interbody fusion (OLIF) has gained increasing popularity recently. However, complications resulting from intraoperative retraction of psoas major (PM) sometimes occur. The aim of this study is to evaluate the degree of PM swelling by developing a scoring system called the Psoas Major Swelling Grade (PMSG), and to investigate the correlation between the PMSG and clinical outcomes after OLIF.

**Methods:**

Patients who underwent L4‐5 OLIF at our hospital from May 2019 to May 2021 were reviewed and all data were recorded. The extent of postoperative PM swelling was determined by calculating the percentage of change in the PM area before and after surgery on MRI and divided into three grades subsequently. Swelling within the range of 0% to 25% was defined as grade I, 25%–50% was grade II, and more than 50% was grade III. All patients were grouped into the new grade system and followed up for at least 1 year, during which the visual analog scale (VAS) and Oswestry disability index (ODI) scores were recorded. Categorical data were analyzed using chi‐square and Fisher's exact tests, while continuous variables were assessed with one‐way ANOVA and paired *t*‐tests.

**Results:**

Eighty‐nine consecutive patients were enrolled in this study, with a mean follow‐up duration of 16.9 months. The proportion of female patients in the PMSG I, II, and III groups was 57.1%, 58.3%, and 84.1%, respectively (*p* = 0.024).  Furthermore, the total complication rate was 43.2% in the PMSG III group, significantly higher than 9.5% and 20.8% in the PMSG I and II groups (*p* = 0.012). The incidence of thigh paraesthesia was also considerably higher in the PMSG III group at 34.1% (*p* = 0.015), compared to 9.5% and 8.3% in the PMSG I and II groups. Among the patients, 12.4% exhibited a teardrop‐shaped PM, with the majority (90.9%) belonging to the PMSG III group (*p* = 0.012). Additionally, the PMSG III group demonstrated a higher estimated blood loss (*p* = 0.007) and significantly worse clinical scores at the 1‐week follow‐up assessment (*p* < 0.001).

**Conclusion:**

PM swelling adversely affects the OLIF prognosis. Female patients with teardrop‐shaped PM are more likely to develop swelling after OLIF. A higher PMSG is associated with a higher complication rate of thigh pain or numbness and worse short‐term clinical outcomes.

## Graphical abstract

Variations of pre‐ and post‐PMCSA in three groups of psoas major swelling grade (PMSG) on MRI (A, B) PMSG I: swelling within the range of 0% to 25%; (C, D) PMSG II: swelling in the range of 25% to 50%; (E, F) PMSG III: swelling more than 50%.

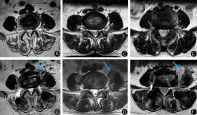

## Introduction

Oblique lumbar interbody fusion (OLIF) is a minimally invasive lumbar fusion procedure that has come into focus in recent years.^1^, ^2^, ^3^ Compared with posterior lumbar interbody fusion (PLIF), anterior lumbar interbody fusion, (ALIF) and extreme lateral interbody fusion (XLIF), OLIF has several advantages including no disruption of the posterior musculoskeletal system, no need to go through the abdominal cavity and no need for neurophysiological monitoring. Besides, faster recovery, shorter duration of hospital stay, less bleeding and higher patient satisfaction after OLIF have also been mentioned in the literature.^1^, ^2^, ^3^, ^4^ However, the incidence of postoperative complications after OLIF was reported to range from 29.9% to 46.2%, which was a concern for spinal surgeons.^2^, ^5^, ^6^ Postoperative complications mainly include transient psoas major (PM) or quadriceps weakness, anterolateral thigh numbness, cage subsidence, vascular injury, and contralateral nerve root injury. Relative to other complications, thigh pain and weakness often occur in the early period of post operation, which may greatly impair the patient's satisfaction.

However, to date, there are few clinical methods for radiologic characteristic assessment of the PM.^6^, ^7^ The correlation between the MRI‐measured PM area and clinical outcomes after OLIF was also rarely reported.^8^ The morphology of the PM can significantly influence the surgical approach for the OLIF procedure. PM swelling may result in pain or numbness in the anterior thigh, potentially compromising short‐term postoperative outcomes.^9^ Consequently, examining the morphology and swelling of the PM is crucial for predicting complications. This study primarily focused on the PM and innovatively introduced a new grading method named Psoas Major Swelling Grade (PMSG), classifying the degree of swelling into three levels—I, II, and III—based on PM swelling severity on MRI scans. To further delve into the variations in clinical efficacy and complications, we also classified the PM morphology into teardrop‐shaped and non‐teardrop‐shaped forms.

The aim of this study encompassed two primary aspects. Firstly, the study aimed to quantify the extent of PM swelling using the three‐level PMSG classification system. This approach provided surgeons with a more precise and comprehensive classification method for evaluating postoperative swelling. Secondly, the study established a connection between PMSG and the severity of short‐term complications, enabling surgeons to predict patients' prognoses in advance and implement early intervention measures accordingly.

## Methods

### Patients

This systematic retrospective study was conducted in the First Affiliated Hospital of Guangzhou University of Chinese Medicine between May 2019 and May 2021. One hundred consecutive patients who underwent OLIF surgery were reviewed, 11 of them were lost to follow‐up due to refusal to cooperate, being out of contact, or death, and a total of 89 patients were included finally. Demographic data, perioperative outcomes, imaging parameters, the visual analog scale (VAS) and Oswestry disability index (ODI) scores were recorded. The postoperative MRI scan time point was the second day after surgery. All patients were followed up for at least 1 year. Institutional review board approval was obtained for this study [No. K(2020)148].

### Inclusion and Exclusion Criteria

Inclusion criteria consisted of the following aspects: (i) age >18 years, underwent L4‐5 OLIF surgery; (ii) mild to moderate spinal stenosis at L4‐5 level, I‐II° lumbar spondylolisthesis, discogenic lumbar pain with or without lower limb radicular pain, which could be significantly relieved after bed rest; (iii) failed conservative treatment for more than 3 months.

Exclusion criteria consisted of the following aspects: (i) a history of previous abdominal or retroperitoneal surgery; (ii) severe bony hyperplasia of the facet joint; (iii) large disc herniation, rupture of the annulus fibrosus, prolapse of the nucleus pulposus; (iv) lesion occupying the spinal canal; (v) comorbidities of serious medical diseases; (vi) severe osteoporosis, scoliosis deformity, spinal infection, tuberculosis, tumor; (vii) lost to follow‐up.

### Measurement Standards of PMSG

**FIGURE 1:**
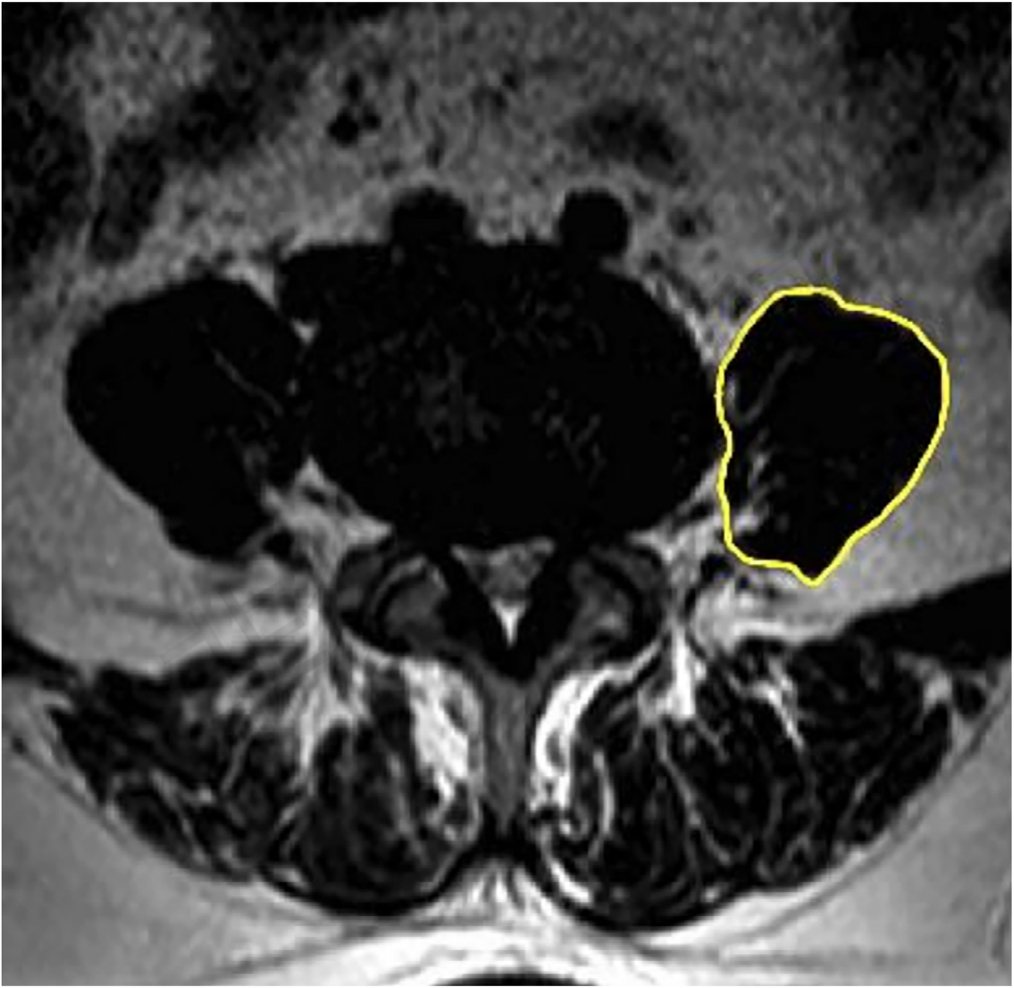
Measurement standard. Measurement of the psoas major cross‐sectional area (PMCSA).

Images of the preoperative and postoperative PM on MRI in the same axial view at L4‐5 level were screenshots captured for the upcoming measurement.^10^, ^11^ The contours of the left PM muscles were carefully outlined, and their areas were measured independently by three senior spine surgeons using ImageJ 1.8.0 (National Institutes of Health, USA) (Figure 1). The proportion of the difference in PM area between preoperative and postoperative measurements to the area of preoperative PM was defined as the degree of PM swelling, which was further divided into three grades.

### Principles and Bases for PMSG

The classification principles and bases for PM swelling shared similarities with paraspinal muscle fat infiltration, as both systems were divided into three levels based on percentage values.^8^ Swelling within the 0%–25% range was defined as grade I, 25%–50% as grade II, and more than 50% as grade III. As Stanuszek et al.^8^ expounded, 25% could be used as a threshold for differentiating the normal (Grade I in our study) and the mild (Grade II in our study) group, and 50% could be a threshold for separating the mild and the severe group (Grade III in our study). Therefore, we believed dividing all patients into three groups was scientific and reasonable.

### Surgical Technique

The patient was placed in the right lateral position under general anesthesia, and the abdominal organs were allowed to dangle naturally under gravity. Cushions were placed under the right thorax and iliac crest, and pillows were placed between the knees. After temporarily fixing the pelvis and knees to stabilize the spine, anteroposterior fluoroscopy was performed to ensure that the spine was not rotated. A 4‐cm‐long incision was made from the left abdominal wall in front of the marker line. The subcutaneous tissue was incised and the external oblique, internal oblique, and transversus abdominis muscles were bluntly separated. The retroperitoneal fat, abdominal organs and vessels were blocked anteriorly and PM was retracted posteriorly by retractors. After reconfirming the correct level under fluoroscopy, the intervertebral space was exposed, the annulus fibrosus was incised, and the nucleus pulposus and endplates were cleared. Next, the contralateral annulus fibrosus was penetrated with a detacher. The size of the cage to be placed was tested with a trial mold. After that, the cage filled with artificial bone was inserted into the intervertebral space and its position was confirmed fluoroscopically. Finally, the incision was rinsed, and the sutures were completed. The patient was then transferred to a prone position for posterior percutaneous screw fixation (PPSF). All procedures were performed by three specially trained spine surgeons.

### Outcome Observations

**FIGURE 2:**
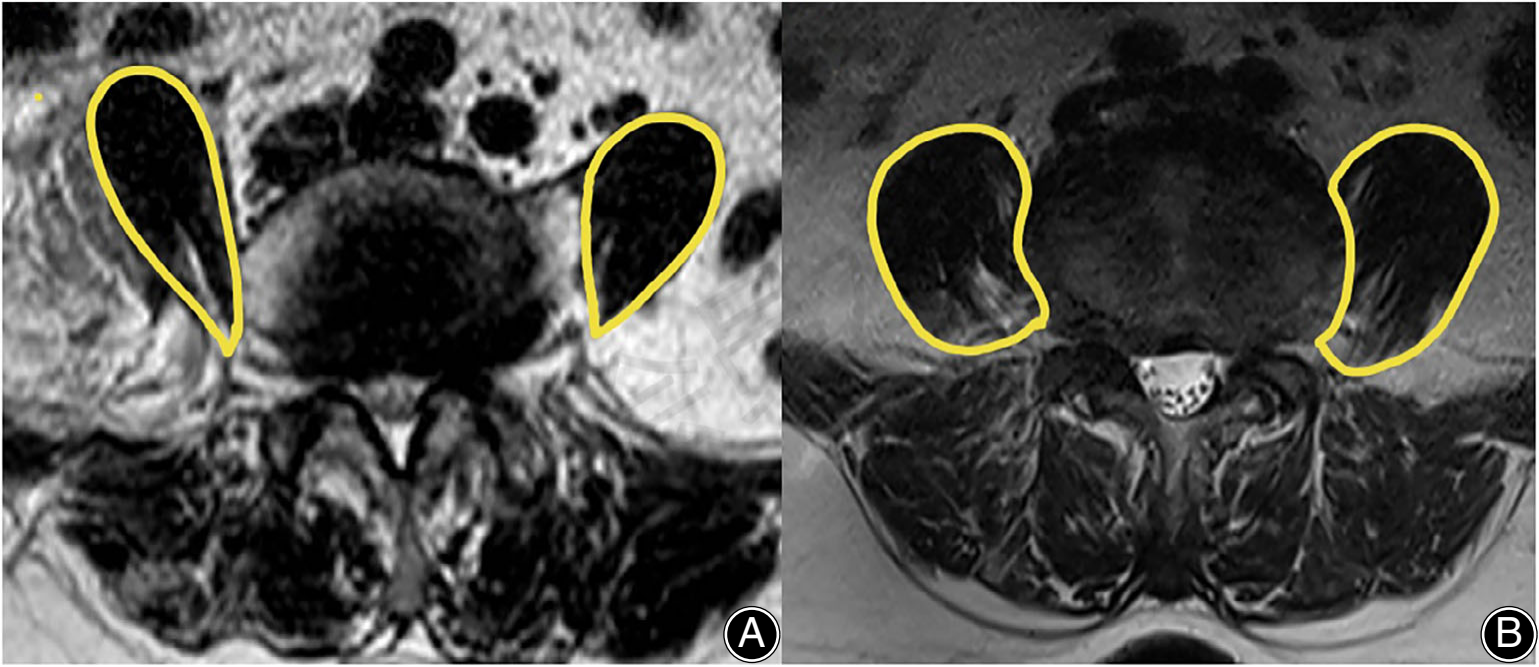
Morphology of psoas major on MRI. (A) Bilateral teardrop‐shaped psoas major; (B) Bilateral normal‐shaped psoas major.

VAS and ODI were used to evaluate clinical efficacy. All scores were recorded at the preoperative, 1‐week postoperative, 3‐months postoperative, 6‐months postoperative, and 12‐months postoperative follow‐ups, respectively. Symptom duration, operation time, estimated blood loss, complications, length of hospital stay, and preoperative PM morphology (teardrop shape or not) were reviewed (Figure 2).^10^

### Statistical Analysis

The data was analyzed by SPSS 23.0 (SPSS Inc., USA) statistical software. The chi‐square test and Fisher's exact test were used to compare categorical variables. One‐way ANOVA was used to analyze numerical variables. Paired *t*‐test was used to compare clinical and imaging outcomes before and after surgery. A *p* value of <0.05 was used as the threshold for significance.

## Results

### Demographic Data

A total of 100 patients initially met the inclusion criteria, and 11 patients were lost to follow‐up. Eighty‐nine patients were finally included in the study. The patients were graded according to the degree of postoperative swelling of the PM. There were 21 cases in PMSG I group, including nine males and 12 females with an average age of 62.33 ± 12.22 years old (mean ± standard deviation). Twenty‐four cases were enrolled in PMSG II group, including 10 males and 14 females with an average age of 59.08 ± 8.26 years. PMSG III group had 44 cases, including seven males and 37 females, with an average age of 60.70 ± 8.51 years. There was a significant difference between PMSG III and the other two groups in respect to gender (*p* = 0.024). No significant difference was observed among the three groups in terms of age, body mass index (BMI), bone mineral density (BMD), symptom duration, and main diagnosis (Table 1).

**TABLE 1:** Summary of patient demographics.

Parameter	PMSG I	PMSG II	PMSG III	Statistic value	*p*‐value
No. of patients	21	24	44		
Age at surgery in years	62.33 ± 12.22	59.08 ± 8.26	60.70 ± 8.51	0.665	0.517
No. of M/F	9/12	10/14^†^	7/37^‡^ ^,^ ^§^	7.456	**0.024**
BMD in g/cm^3^	−2.25 ± 0.94	−2.07 ± 0.68	−2.15 ± 0.77	0.291	0.748
BMI in kg/m^2^	24.12 ± 2.30	23.71 ± 2.57	23.62 ± 2.99	0.241	0.786
Symptom duration in years	5.64 ± 4.11	4.46 ± 3.34	5.13 ± 3.58	0.600	0.551
Main diagnosis				0.715	0.950
Lumbar spinal stenosis	4	5	11		
Spondylolisthesis, instability	13	16	26		
Discogenic lumbago	4	3	7		

*Note*: Numerical variables are expressed as mean ± SD. Categorical variables are expressed as no.

Abbreviations: BMI, body mass index; BMD, bone mineral density; SD, standard deviation.

^†^ *p* > 0.05, compared with PMSG I.

^‡^ *p* < 0.05, compared with PMSG I.

^§^ *p* < 0.05, compared with PMSG II.

### Perioperative Data

PMSG III group showed more estimated blood loss than PMSG I and II groups (*p* = 0.007), and there was no significant difference between PMSG I and II groups. The duration of surgery (excluding PPSF) was 39.43 ± 3.46, 38.96 ± 3.43, and 39.14 ± 3.65 min in each group, respectively, with no statistically significant difference. The length of hospital stay was 6.10 ± 1.73, 6.33 ± 1.93, and 6.77 ± 1.57 days in each group, respectively, with no statistically significant difference.

### Complications

As for complications, two (9.52%), five (20.83%), and 19 (43.18%) cases were reported in three groups, respectively. The total complication rate in PMSG III group was significantly higher than PMSG I group (*p* = 0.012), while there was no statistically significant difference among other groups. Two (9.52%) patients in PMSG I group, two (8.33%) in PMSG II group, and 15 (34.09%) in PMSG III group reported thigh pain and numbness or hip flexion weakness after operation immediately. Evidently, PMSG III group showed a much higher incidence of iliopsoas symptoms than the other two groups (*p* = 0.015). Sympathetic chain injury was reported by one patient in PMSG III and PMSG II group, respectively, with no statistically significant difference. In addition, three (6.82%) and two (8.33%) patients in PMSG III group and PMSG II group had cage subsidence and endplate fracture, with no statistically significant difference. All patients who developed iliopsoas symptoms such as thigh pain and numbness or hip flexion weakness were cured by oral NSAIDs or resolved on their own during follow‐up. Patients with cage subsidence did not develop severe clinical outcomes during follow‐up. No complications such as permanent neurological impairment or ureteral or peritoneal injury were observed throughout the whole follow‐up (Table 2).

**TABLE 2:** Perioperative results.

Parameter	PMSG I	PMSG II	PMSG III	Statistic value	*p*‐value
Estimated blood loss in ml	56.19 ± 19.62	66.25 ± 19.52^†^	73.86 ± 21.91^‡^ ^,^ ^§^	5.220	**0.007**
Operation time in minutes	39.43 ± 3.46	38.96 ± 3.43	39.14 ± 3.65	0.100	0.905
Length of hospital stay in days	6.10 ± 1.73	6.33 ± 1.93	6.77 ± 1.57	1.264	0.288
Complications					
Total complication rate	2 (9.52)	5 (20.83)^†^	19 (43.18)^‡^ ^,^ ^§^	8.904	**0.012**
Thigh pain/numbness, hip flexion weakness	2 (9.52)	2 (8.33)^†^	15 (34.09)^‡^ ^,^ ^ψ^	8.425	**0.015**
Sympathetic chain injury	0 (0)	1 (4.17)	1 (2.27)	0.885	0.642
Cage subsidence/endplate injury	0 (0)	2 (8.33)	3 (6.82)	1.703	0.427

*Note*: Numerical variables are expressed as mean ± SD. Complications are expressed as no. (%).

^†^ *p* > 0.05, compared with PMSG I.

^‡^ *p* < 0.05, compared with PMSG I.

^§^ *p* > 0.05, compared with PMSG II.

^ψ^ *p* < 0.05, compared with PMSG II.

### Imaging Parameters

**FIGURE 3:**
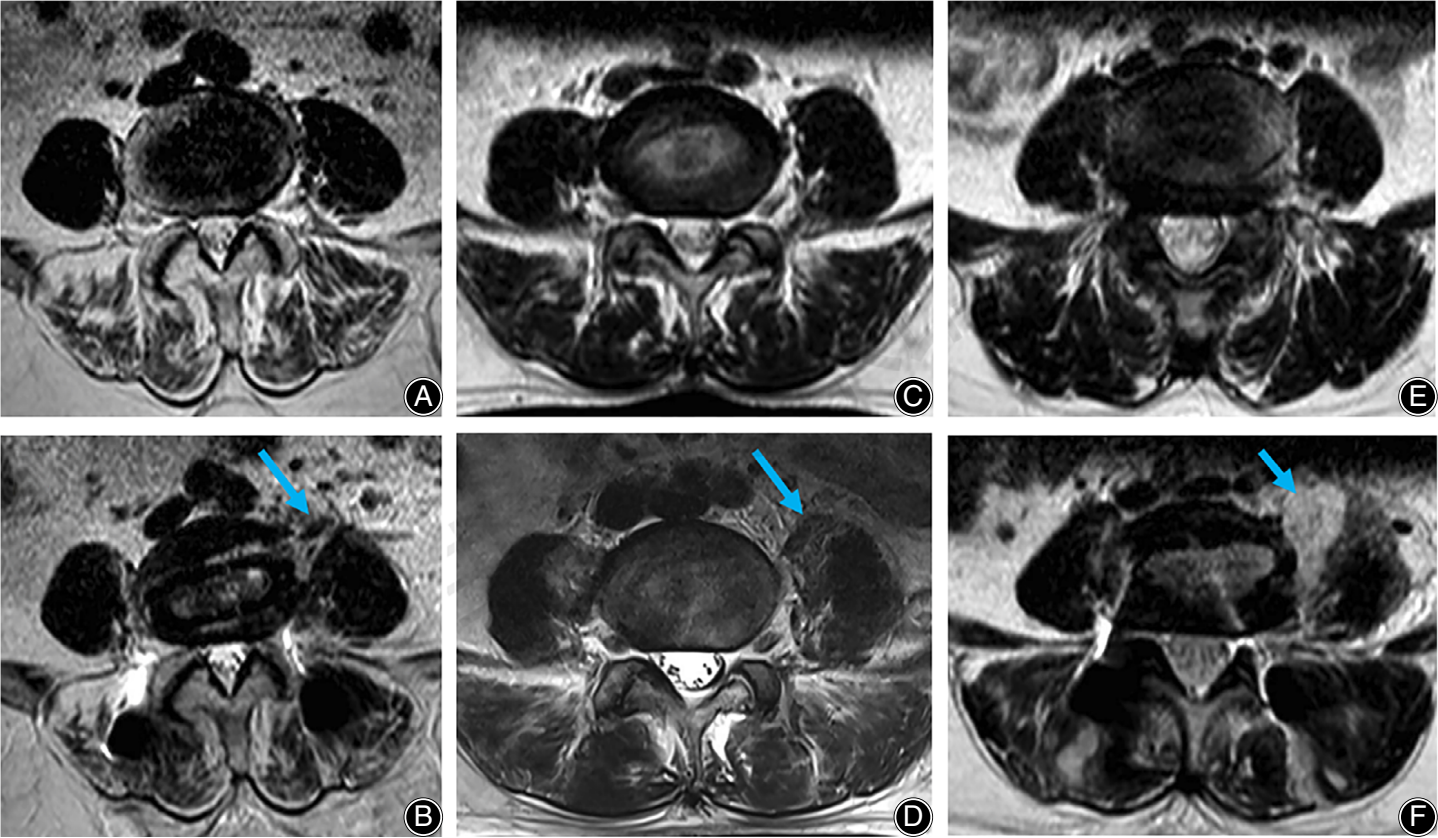
Variations of pre‐ and post‐PMCSA in three groups of psoas major swelling grade (PMSG) on MRI. (A, B) PMSG I: swelling within the range of 0–25%; (C, D) PMSG II: swelling in the range of 25% to 50%; (E, F) PMSG III: swelling more than 50%.

Regarding the imaging parameters, the preoperative PM cross‐sectional area (PMCSA) at the L4‐5 level was 1083.92 ± 269.41, 1046.59 ± 187.40, and 1018.58 ± 226.05 mm^2^ in each group, respectively, with no statistical significance. The postoperative PMCSA in PMSG III group was significantly larger than that in PMSG II group (*p* < 0.05) and PMSG I group (*p* < 0.001), whereas there was no significant difference between the PMSG I and II groups. The postoperative swelling percentage of the PM was also significantly higher in PMSG III group than in the other two groups (*p* < 0.001), while the percentage between the PMSG II and I groups was also statistically different (*p* < 0.001). As for the preoperative PM muscles being teardrop‐shaped or not, none of them were found in PMSG I group, while one and 10 cases were found in the PMSG II and III groups, respectively, with a statistically significant difference (*p* = 0.012) (Table 3). Figure 3 showed the variations between the preoperative and postoperative PMCSA among the three groups.

**TABLE 3:** Imaging parameters.

Parameter	PMSG I	PMSG II	PMSG III	Statistic value	*p*‐value
Pre‐op PMCSA in mm^2^	1083.92 ± 269.41	1046.59 ± 187.40	1018.58 ± 226.05	0.594	0.555
Post‐op PMCSA in mm^2^	1242.42 ± 331.01	1448.52 ± 269.97*	1670.93 ± 372.15^†^ ^,^ ^‡^	11.967	**<0.001**
Swelling in percent	14.38 ± 4.70	38.42 ± 7.11^†^	64.16 ± 6.91^†^ ^,^ ^§^	433.443	**<0.001**
Teardrop‐shaped psoae	0 (0)	1 (4.17)*	10 (22.73)^Φ^ ^,^ ^‡^	8.815	**0.012**

*Note*: Numerical variables are expressed as mean ± SD. Teardrop‐shaped psoae are expressed as no. (%). Bold value indicates the significant value (*p* < 0.05).

Abbreviations: pre‐op, preoperative; post‐op, postoperative.

* *p* > 0.05, compared with PMSG I.

^†^ *p* < 0.001, compared with PMSG I.

^‡^ *p* < 0.05, compared with PMSG II.

^§^ *p* < 0.001, compared with PMSG II.

^Φ^ *p* < 0.05, compared with PMSG I.

### Clinical Outcomes

Clinical scores were displayed in Table 4. All patients showed significant improvement in all scores during the follow‐up period. Notably, all scores were worse in PMSG III group than in the other two groups at 1 week post operation (*p* < 0.001), whereas no difference was observed between PMSG I and II groups.

**TABLE 4:** Clinical outcomes.

Parameter	PMSG I	PMSG II	PMSG III	Statistic value	*p*‐value
VASb					
Pre‐op	7.24 ± 1.00	6.83 ± 1.24	6.84 ± 1.01	1.114	0.333
1‐week post‐op	2.52 ± 0.75	2.71 ± 0.81*	3.30 ± 0.59^†^ ^,^ ^‡^	10.881	**<0.001**
3‐months post‐op	1.19 ± 0.68	1.21 ± 0.42	1.32 ± 0.47	0.594	0.554
6‐months post‐op	0.43 ± 0.51	0.46 ± 0.51	0.48 ± 0.51	0.066	0.936
12‐months post‐op	0.24 ± 0.44	0.29 ± 0.46	0.36 ± 0.49	0.548	0.580
VASl					
Pre‐op	7.43 ± 1.17	6.92 ± 1.21	7.00 ± 1.10	1.322	0.272
1‐week post‐op	2.67 ± 0.80	2.88 ± 0.45*	3.32 ± 0.47^†^ ^,^ ^‡^	11.189	**<0.001**
3‐months post‐op	1.29 ± 0.72	1.33 ± 0.48	1.41 ± 0.50	0.391	0.677
6‐months post‐op	0.52 ± 0.51	0.54 ± 0.51	0.50 ± 0.51	0.055	0.947
12‐months post‐op	0.33 ± 0.48	0.42 ± 0.50	0.48 ± 0.51	0.597	0.553
ODI					
Pre‐op	74.33 ± 7.88	73.88 ± 3.71	74.82 ± 6.49	0.181	0.835
1‐week post‐op	36.05 ± 3.87	37.25 ± 3.22*	40.48 ± 4.11^†^ ^,^ ^‡^	11.409	**<0.001**
3‐months post‐op	21.67 ± 4.29	22.21 ± 4.75	22.77 ± 3.06	0.605	0.549
6‐months post‐op	9.10 ± 1.81	9.25 ± 2.07	9.45 ± 1.61	0.308	0.736
12‐months post‐op	1.05 ± 0.87	1.17 ± 0.76	1.36 ± 1.03	0.911	0.406

*Note*: Bold value indicates the significant value (*p* < 0.05).

Abbreviations: pre‐op, preoperative; post‐op, postoperative; VASb, visual analog scale of back; VASl, visual analog scale of leg; ODI, Oswestry disability index.

* *p* > 0.05, compared with PMSG I.

^†^ *p* < 0.001, compared with PMSG I.

^‡^ *p* < 0.05, compared with PMSG II.

### Typical Case Presentation

**FIGURE 4:**
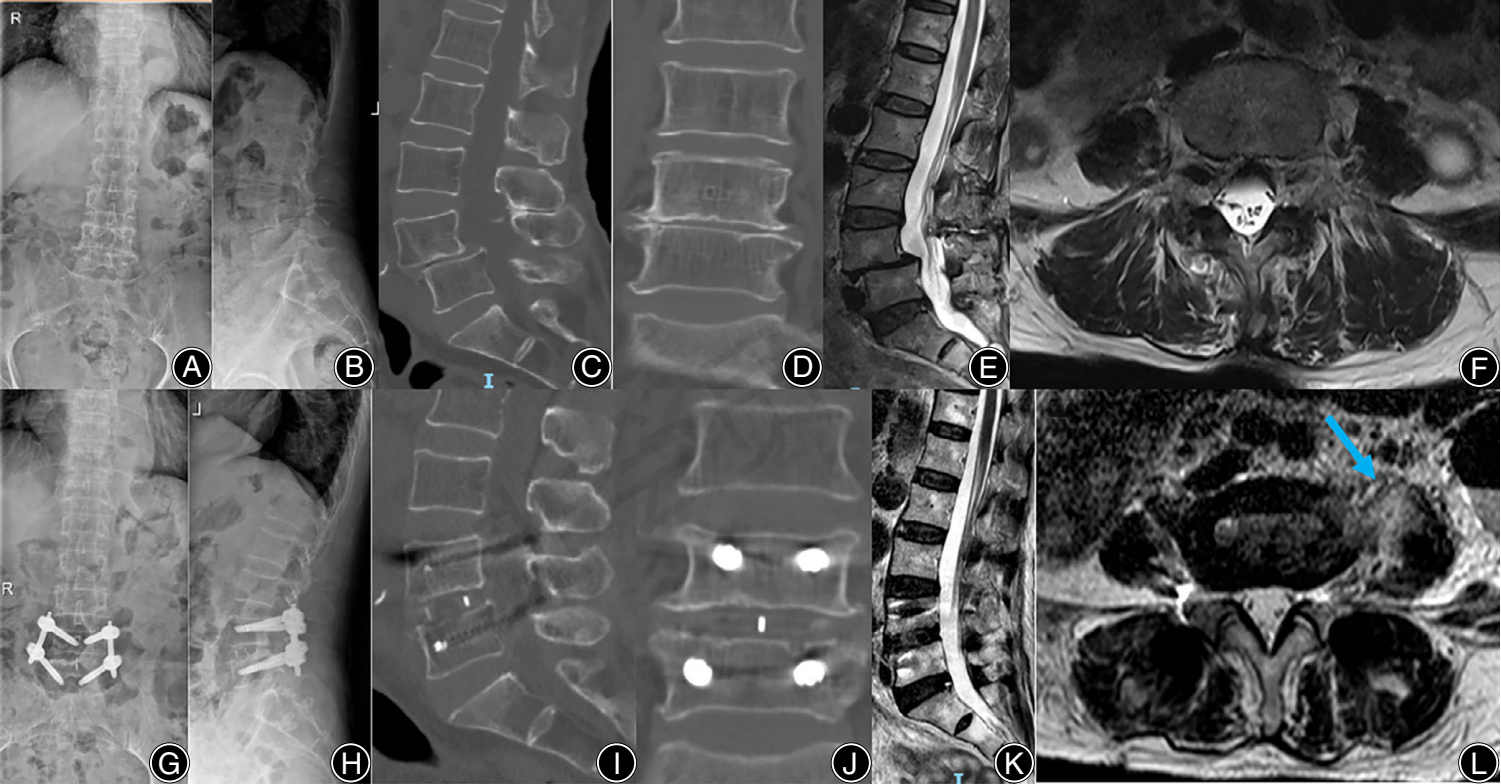
Typical case. A 72‐year‐old woman, presented with pain and numbness in both lower extremities for 6 years and worsened for 2 weeks. (A, B) Preoperative lumbar X‐ray; (C, D) Preoperative lumbar CT; (E, F) Preoperative lumbar MRI. Preoperative images showed the L4 spondylolisthesis and stenosis at L4‐5; (G, H) Postoperative lumbar X‐ray showed L4 was reduced; (I, J) Postoperative lumbar CT showed distraction of disc height; (K, L) Postoperative lumbar MRI showed satisfactory decompression and a huge swelling of the left psoas major (arrow).

**FIGURE 5:**
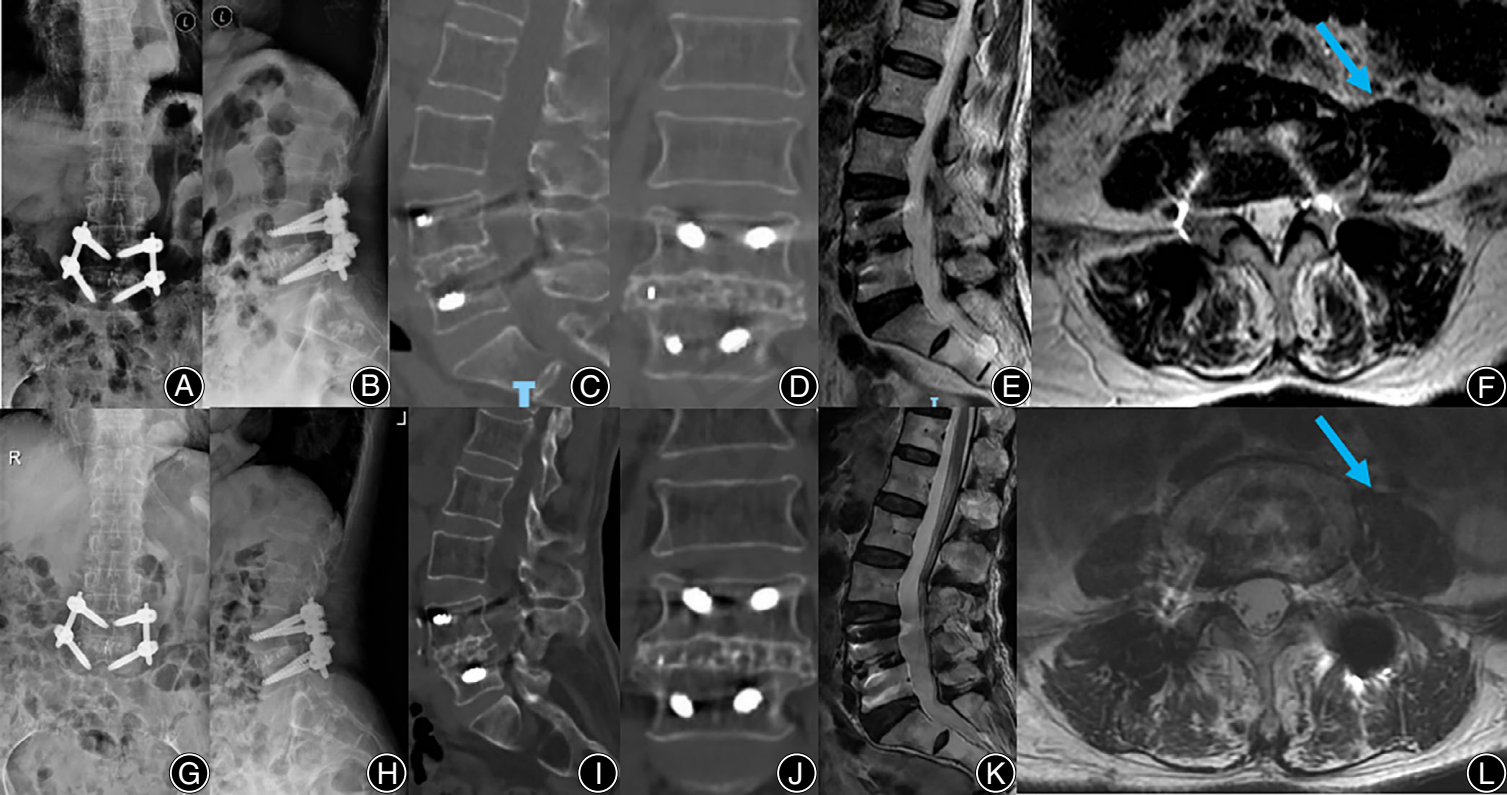
Follow‐up of the typical case. (A–F) Lumbar X‐ray, CT, and MRI at 1‐year follow‐up; (G–L) Lumbar X‐ray, CT, and MRI at 2‐year follow‐up. All follow‐up data showed solid fusion with no cage subsidence or displacement at L4‐5. Notably, compared with immediately post operation, swelling of the left psoas major reduced significantly (arrow), and its area recovered after 1‐year follow‐up.

A typical case was shown in Figures 4 and 5. The patient with PMSG III had a PM that had returned to preoperative size at 1‐year follow‐up. The rest of the patients had a similar morphology change of PM muscles as this woman at 1‐year follow‐up. This was consistent with our clinical outcomes, which meant that no matter how severe the short‐term postoperative complications were, they did not affect the patient's quality of life at 1‐year follow‐up.

## Discussion

### Main Findings

This study has evaluated the extent of PM swelling and its association with clinical outcomes and complications following OLIF surgery. Ascertained from MRI scans on the second day after surgery, PMSG enabled surgeons to assess short‐term clinical effectiveness, anticipate short‐term complications, and tailor treatment strategies accordingly. It was particularly crucial for patients classified as PMSG III to consider early initiation of intensified anti‐inflammatory and dehydration measures or physiotherapy as a prophylactic approach. Moreover, the study highlighted the relevance of anatomical gender differences and morphological variations in teardrop shapes to higher PMSG, suggesting that surgeons should exercise increased caution during preoperative evaluations for female patients presenting with PM variants. In summary, as a rapidly obtainable indicator on the second day following surgery, PMSG could effectively predict short‐term clinical outcomes and complications, guiding personalized prophylactic measures and benefiting clinical practice.

### Practicality and Measurement Accuracy

The practicality of PMSG stemmed from its simplicity, requiring only PM areas before and on the second day after surgery to predict short‐term complications without necessitating additional complex parameter measurements or calculations. Moreover, the learning curve for mastering the measurement method took less than an hour for surgeons, thus offering both simplicity and cost‐effectiveness. The measurement accuracy was ensured using MRI images as the original data source. MRI is one of the most precise and clear imaging techniques available for muscle evaluation, providing accurate and reliable image information based on its underlying imaging principles.

### Female Patients in PMSG III Group

We found that the proportion of females was much higher in PMSG III group than in the other two groups (*p* = 0.024). A study conducted by Liu et al. revealed that females were not suitable for OLIF at the L4‐5 level because their physiological anatomy of the abdominal aortic bifurcation and iliac vein confluence was located in front of the L4‐5 disc, which caused high risks of vascular injury.^12^ Another study by Chen et al. showed that the width and thickness of PM muscles at L4‐5 level were greater than those at L3‐4 and L2‐3 levels.^13^ Consequently, we believed that during OLIF surgery in female patients, the surgeons would overstretch the PM to obtain a better surgical field. Meanwhile, the PM muscles in males were wider and thicker than in females, which meant females were more intolerant of muscle stretching. It was the reason why women were more involved in PMSG III group than men. In the perioperative data, the estimated blood loss was significantly different between PMSG III and I groups (*p* = 0.007), suggesting that the amount of bleeding was probably an important factor affecting the swelling of PM.

### Patients with Higher PMSG Suffered More Complications

Although OLIF does not cause damage to the posterior muscles, it still requires stretching of the lateral abdominal vessels, nerves, and muscles to obtain an excellent surgical view, resulting in some damage and corresponding postoperative complications, such as vascular injury, sympathetic chain injury, and muscle swelling.^14^, ^15^, ^16^ Our results showed that patients in PMSG III group suffered more postoperative complications than those in PMSG II and I group with a significant difference (*p* = 0.015). Thigh pain and numbness or hip flexion weakness were more common and only very few patients had sympathetic chain injury and cage subsidence. Several studies have shown that the lumbar plexus was close to surgical access at L4‐5 level, which meant it was in a particularly dangerous condition during transabdominal surgery.^17^, ^18^, ^19^, ^20^ A study conducted by Kirchmair et al. also showed that most of the lumbar plexus were located inside PM muscles in 96.8% of patients, and only two (2/63) were located posterior to the PM muscles in their study.^21^ Both cases could lead to compression of the lumbar plexus, resulting in symptoms such as sensory numbness or iliopsoas asthenia when PM muscles were swollen by overstretching. The greater the stretching of the PM during surgery, the higher the degree of swelling observed postoperatively. The data indicated that patients with higher PMSG experienced more significant intraoperative damage to their PM muscles, which might account for their poorer short‐term clinical outcomes. It was plausible that the adverse impacts of these short‐term complications counterbalance the positive effects of the surgery itself, further confirming that the higher the PMSG, the more severe the compression of the lumbar plexus and the higher the possibility that iliopsoas symptoms might occur.

### Teardrop‐Shaped PM Muscles Were more Susceptible to Swelling

Different preoperative morphology of PM could lead to different PMSG after surgery. A kind of PM variant known as the teardrop‐shaped PM has been reported, which might affect clinical efficacy.^10^, ^11^ Our study found that 90.9% of teardrop‐shaped PM muscles were in PMSG III group, which was far greater than the other group (*p* = 0.012). Ng et al. concluded that 10.5% of patients did not have a natural oblique corridor and 20% of patients undergoing OLIF had a structural abnormality of the PM muscles.^22^ We believe that it was this PM variant that narrowed the surgical corridor, entailing greater intraoperative retraction on the PM to gain a clear surgical field. This preoperative physiologic difference affected the surgical approach and might be responsible for the increased postoperative swelling of the PM.

### The Potential for Additional Subdivision of PMSG

PMSG III group exhibited significantly poorer efficacy outcomes at 1‐week follow–up after the operation, indicating that high PMSG levels might adversely affect short‐term clinical efficacy (*p* < 0.001). However, no statistically significant differences were observed between the PMSG I and II groups. Further more, no significant variations were found among the demographics, perioperative results, and imaging parameters between the PMSG I and II groups. Hence, it was deemed reasonable to combine patients from the PMSG I and II groups and redefine the PMSG category. For future studies, a threshold of 50% could be utilized to reclassified PMSG, with PMSG values below 50% falling under the general group and values equal to or exceeding 50% categorized as the overswelling group. This classification could serve as a basis for determining whether additional postoperative anti‐inflammatory and dehydration treatments are necessary.

### Limitations and Strengths of the Study

The strengths of the study could be highlighted in two primary aspects. First, in terms of postoperative evaluation, this study introduced an innovative PMSG classification system to grade the extent of postoperative PM swelling. This classification not only assisted surgeons in assessing short‐term clinical outcomes following OLIF surgery but also aided in predicting short‐term complications. Second, concerning preoperative evaluation, the study identified risk factors correlated with high PMSG. A more comprehensive preoperative assessment and gentler intraoperative traction were warranted for female patients and those exhibiting teardrop‐shaped PM muscles.

However, there were also several limitations of this research. First, this study was retrospective and was subject to some selection and recall bias. Second, the PM swelling was indistinguishable from hematoma or edema when measuring because they were miscellaneous and blurred on MRI. Third, with small sample size and short follow‐up time, longer follow‐up or prospective studies with a larger sample size are needed in the future.

### Conclusion

Postoperative swelling of the PM affected short‐term clinical outcomes. The higher the PMSG, the more chance of blood loss and higher complication rate of thigh pain or numbness. Women and patients with teardrop‐shaped PM muscles were more likely to develop PM swelling after OLIF.

## AUTHOR CONTRIBUTIONS

Zefeng Song, Wanyan Chen, and Jingjing Tang contributed to the study conception and design. Material preparation, data collection, and analysis were performed by Zefeng Song, Wanyan Chen, Xingda Chen, Shaohao Lin, Xiaowen Wang, and Peng Zhang. The first draft of the manuscript was written by Wanyan Chen, Zefeng Song, Guangye Zhu, and Zelin Zhou. Xiang Yu, Hui Ren, Jianchao Cui, De Liang, and Xiaobing Jiang commented on previous versions of the manuscript. All authors read and approved the final manuscript.

## FUNDING INFORMATION

This research received no specific grant from any funding agency in the public, commercial, or not‐for‐profit sectors, and the authors declare that there is no conflict of interest.

## CONFLICT OF INTEREST STATEMENT

The authors declare that there is no conflict of interest. All the authors had met the authorship criteria according to the latest guidelines of the International Committee of Medical Journal Editors, and they are all in agreement with the manuscript.

## ETHICAL STATEMENT

This study was performed in line with the principles of the Declaration of Helsinki. Approval was granted by the Ethics Committee of the First Affiliated Hospital of Guangzhou University of Chinese Medicine [No. K（2020）148] and the ethics committee agreed to waive informed consent.
